# Factors affecting the occupancy of sloth bear and its detection probability in Parsa–Koshi Complex, Nepal

**DOI:** 10.1002/ece3.10587

**Published:** 2023-10-03

**Authors:** Hari Prasad Sharma, Hem Bahadur Katuwal, Bishnu Prasad Bhattarai, Shivish Bhandari, Dipendra Adhikari, Bishnu Aryal, Krishna Tamang, Amrit Nepali, Sabin KC, Bashu Dev Baral, Surya Devkota, Sabina Koirala, Dev Narayan Mandal, Sandeep Regmi

**Affiliations:** ^1^ Central Department of Zoology, Institute of Science and Technology Tribhuvan University Kirtipur, Kathmandu Nepal; ^2^ Nepal Zoological Society Kirtipur, Kathmandu Nepal; ^3^ Center for Integrative Conservation, Xishuangbanna Tropical Botanical Garden Chinese Academy of Sciences Mengla China; ^4^ Department of Biology Morgan State University Baltimore Maryland USA; ^5^ Nepal Conservation and Research Center Chitwan Nepal; ^6^ Mithila Wildlife Trust Janakpurdham, Dhanusa Nepal

**Keywords:** camera traps, lowland, *Melursus ursinus*, occupancy, Parsa–Koshi Complex, sloth bear

## Abstract

Understanding factors associated with coexistence of human and wildlife in human‐dominated landscapes is crucial for effective species conservation. Among the wildlife species, the sloth bears *Melursus ursinus* are found both inside and outside the protected areas of Nepal, and with increasing cases of human and bear conflicts in both areas. This highlights the necessity for a comprehensive understanding of anthropogenic and ecological factors that affect the occurrence of sloth bear. The understanding of these factors is important for its coexistence and conservation in human‐dominated areas through establishing management and conservation action plan. We studied the sloth bear's occupancy and their coexistence in human‐dominated environments with other large predators in the Parsa–Koshi Complex of Nepal using camera traps from December 2022 to March 2023. We identified the occupancy and detection probability of the sloth bear as 0.12 and 0.31, respectively. Our analysis reveals a positive relationship between sloth bear occurrence and the presence of large predators (*βpredators* = 3.104 ± 0.968), such as tigers (*Panthera tigris*) and leopards (*Panthera pardus*), as well as the number of humans detected (*βhuman* = 1.428 ± 1.216) and canopy cover percentage (*βcc* = 1.002 ± 0.737). However, the number of livestock detected shows a negative interaction with the occurrence of sloth bears (*βlivestock* = −2.240 ± 1.467). There was insignificant interaction between sloth bear occupancy and distance to human settlements, roads, and water bodies. These findings underscore the complex dynamics between sloth bears, humans, large predators, and livestock in human‐dominated landscapes. To ensure the long‐term survival of sloth bear populations and promote species conservation, comprehensive conservation strategies that account for both ecological and socio‐economic factors are essential.

## Graphical abstract

The findings underscore the complex dynamics between sloth bears, humans, large predators, and livestock in human‐dominated landscapes. To ensure the long‐term survival of sloth bear populations and promote species conservation, comprehensive conservation strategies that account for both ecological and socio‐economic factors are essential.

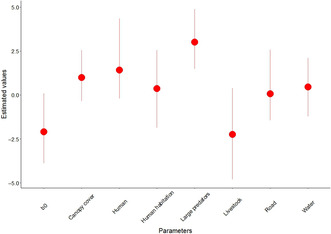

## 1 INTRODUCTION

Effective species conservation in human‐dominated landscapes relies on a comprehensive understanding of species coexistence (Tarjuelo et al., 2014). Such studies provide valuable insights into the interactions between different species in terms of space utilization and resource allocation within similar habitats (Gehr et al., 2017; Li et al., 2019; Milleret et al., 2018; Oriol‐Cotterill et al., 2015; Suraci et al., 2020). The coexistence of species provides detailed information on the complex ecological relationships that exist within ecosystems (Bhandari et al., 2022; Levine et al., 2017; Suraci et al., 2020). In human‐dominated landscapes, species coexistence helps to understand how human activities, such as urbanization, agriculture, and infrastructure development, impact species behavior and their ability to coexist in these environments (Bhandari et al., 2022; Levine et al., 2017) and to some extent triggers of human–wildlife conflicts (Pant et al., 2023). This knowledge is essential for the implementation of sustainable land use practices that minimize negative impacts on species and promote harmonious coexistence between humans and other species (Ahmadi et al., 2013; Carricondo‐Sanchez et al., 2019; Sillero‐Zubiri & Laurenson, 2001; Soga & Gaston, 2020). Furthermore, species interactions in human‐dominated landscapes allow us to develop strategies for biodiversity protection and conservation (Ahlering et al., 2013; Baral et al., 2022; Karanth & DeFries, 2010; Miller, 2005; Persha et al., 2010). By comprehending how different species coexist, people can identify key factors that contribute to their successful coexistence and design conservation measures to ensure the long‐term survival of various species (Gehr et al., 2017; Miller, 2005; Oriol‐Cotterill et al., 2015; Pant et al., 2023).

Occupancy analysis is a reliable and widely used technique for investigating species interactions in relation to their ecological and anthropogenic variables (Devarajan et al., 2020; MacKenzie et al., 2002, 2017; Nichols et al., 2007; Regmi et al., 2023; Sharma et al., 2023). The occupancy model estimates species distribution based on the presence or absence of species in specific areas (MacKenzie, 2005; Nichols et al., 2007). This approach provides valuable insights into habitat quality and suitability. Studying the occupancy of carnivores is particularly informative as it sheds light on predator–prey relationships (Ghoshal et al., 2019), trophic cascades (Jones et al., 2016; Justa & Lyngdoh, 2023), and the overall functioning of ecosystems. Understanding how carnivores occupy and utilize habitats provides critical information for making informed decisions related to wildlife management, and mitigating human–wildlife conflicts (MacKenzie, 2005; Nichols et al., 2007). Moreover, the occupancy method assists in identifying important habitat areas for carnivores and assessing their population dynamics, helps to evaluate the impacts of human activities on carnivore occupancy, and informs strategies for effective land management (Heinrichs et al., 2010; Van der Weyde et al., 2022). By understanding how carnivores interact with their environment, people can develop appropriate measures to conserve and manage these species while minimizing conflicts with human activities (Das et al., 2014; Puri et al., 2023; Van der Weyde et al., 2022).

The sloth bear *Melursus ursinus* is distributed in the Indian Subcontinent, including countries like Nepal, India, and Sri Lanka (Dharaiya et al., 2020; Joshi et al., 1997; Puri et al., 2015; Rather et al., 2020; Ratnayeke et al., 2007; Ratnayeke & Van Manen, 2012; Rot et al., 2023). The species is globally vulnerable and nationally endangered in Nepal with a declining population and threats to habitat primarily due to human‐induced activities (Dharaiya et al., 2020; Joshi et al., 1995). The sloth bear is distributed below 2000 m above the sea level (asl) (Dharaiya et al., 2020), and in Nepal, its distribution is limited to lowland regions (Jnawali et al., 2011) This range encompasses diverse landscapes, including tropical and sub‐tropical forests, broad‐leaved forests, and rhododendron forests, covering both protected and non‐protected areas, including grasslands, thorn scrub, and sal (*Shorea robusta*) forest (Dharaiya et al., 2020; Garshelis, Joshi, & Smith, 1999; Garshelis, Joshi, Smith, et al., 1999). The occurrence of sloth bears in Nepal has been documented in protected areas, such as Chitwan National Park, Parsa National Park, Banke National Park, and Bardia National Park (Garshelis, Joshi, & Smith, 1999; Garshelis, Joshi, Smith, et al., 1999; Ghimire & Thapa, 2014; Joshi et al., 1995; Laurie & Seidensticker, 1977; Pokharel et al., 2022) as well as in various community forests situated in the lowland and hure regions of Nepal (Pokharel et al., 2022; Pokhrel, 2007). However, limited information is available regarding their distribution and threats to their conservation in their potential distribution areas, mainly lowlands (Garshelis, Joshi, & Smith, 1999; Garshelis, Joshi, Smith, et al., 1999; Pokharel et al., 2022). Therefore, it is crucial to collect more comprehensive information on sloth bear populations and the specific conservation challenges they encounter in Nepal's lowland regions. Understanding their occurrence, abundance, and challenges will facilitate the development of species‐focused conservation strategies (Joshi et al., 1995; Laurie & Seidensticker, 1977). By focusing on these potential distribution areas, people can strengthen conservation initiatives aimed at safeguarding sloth bears and their habitats in Nepal.

Our study focused on the Parsa–Koshi Complex (PKC) in Nepal, which encompasses two protected areas: Parsa National Park (PNP) in the west and Koshi Tappu Wildlife Reserve (KTWR) in the east. A large human‐dominated area lies in between these two protected areas, measuring a span of around 500 km. Additionally, the landscape includes various community forests, religious forests, and corridor forests in the lowland regions (Chaudhary et al., 2016; DFRS, 2015; Dhakal & Masuda, 2009; Nagendra, 2002; Timilsina & Heinen, 2008). The PKC is recognized as a significant habitat for the sloth bear; however, increasing urbanization, deforestation, and various anthropogenic pressures, including land use and land cover change (Kafle et al., 2023) are causing a decline in the sloth bear's habitat as well as other large predators' habitats and affecting their activities and behavior (Bhandari et al., 2022; Ratnayeke et al., 2014; Smith & Mishra, 1992). Rapid population growth and industrialization in Nepal's tarai have resulted in extensive deforestation and widespread developmental activities in natural forest areas (Bajracharya, 1983; Chakraborty, 2001; Chaudhary et al., 2016). However, there has been limited research on sloth bears in these regions (Joshi et al., 1995; Laurie & Seidensticker, 1977; Rai et al., 2022) and understanding the anthropogenic and ecological factors influencing sloth bear habitat ecology and occupancy in this landscape is crucial for species conservation in the PKC. In this study, we aimed to address the question of how anthropogenic and ecological variables, such as the presence of large predators such as tigers (*Panthera tigris*) and leopards (*Panthera pardus*), are associated with the occupancy of sloth bears in human‐dominated environments. We hypothesize that anthropogenic and ecological factors will have a negative and positive influence on sloth bears' occupancy, respectively, in their habitats. By investigating these relationships, we can gain insights into the factors influencing sloth bear populations in the PKC and carry conservation efforts to protect this species in the face of increasing human activities and habitat degradation in the PKC.

## 2 MATERIALS AND METHODS

### 2.1 Study area

**FIGURE 1:**
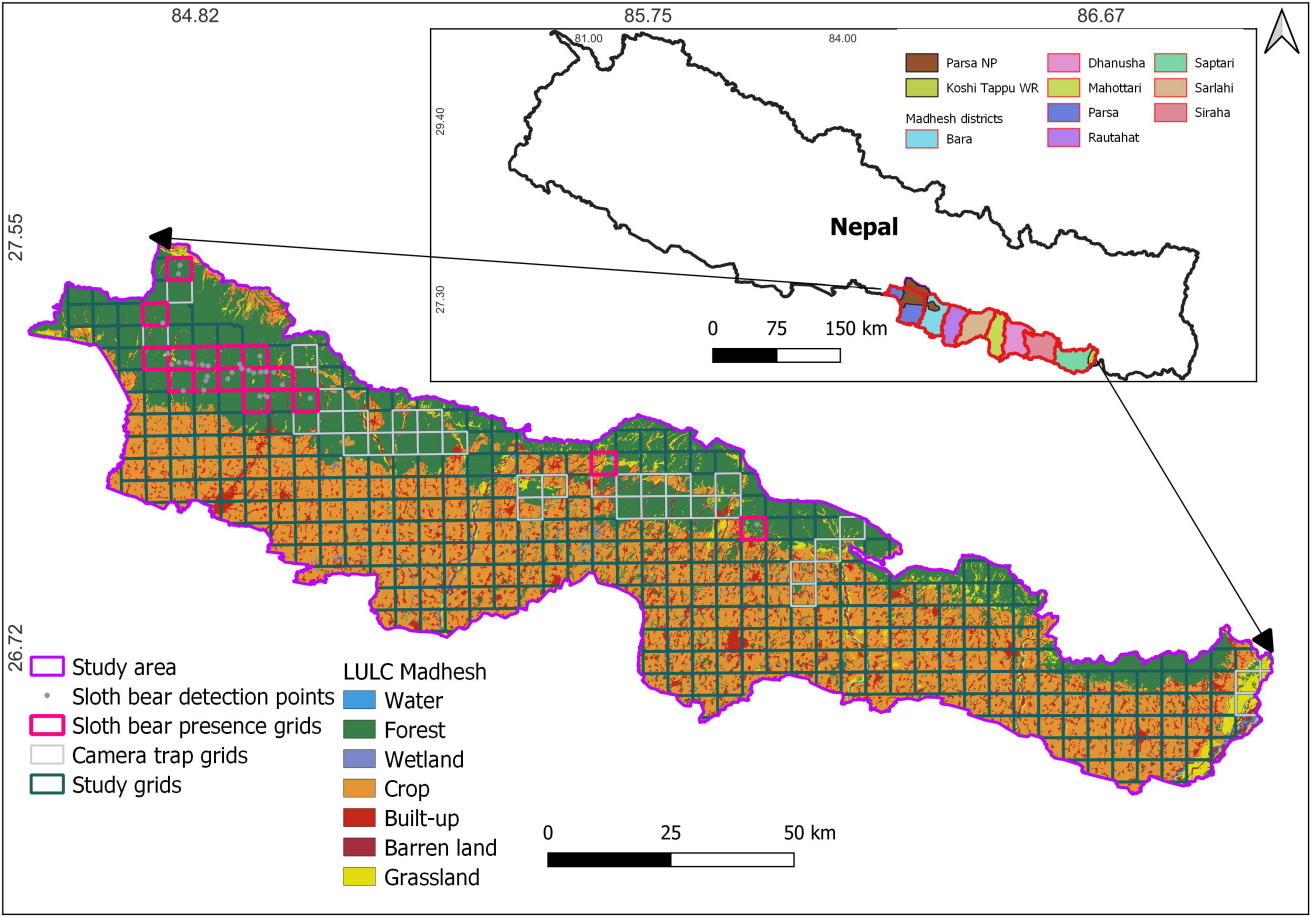
Sloth bear study area in Parsa–Koshi Complex, Nepal. Protected areas and district administrative boundaries are illustrated in the insect.

We conducted this study within the geographical boundaries of the PKC, which encompasses the regions between PNP in the west and KTWR in the west, situated in the lowlands of Nepal at the Madhesh Province (Figure 1). It comprises an area of 9661 km^2^ with a unique and diverse landscape that holds great ecological importance. With an altitude ranging from 80 to 800 m, it spans across eight districts in lowland Nepal, including Bara, Parsa, Rautahat, Sarlahi, Mahottari, Dhanusha, Siraha, and Saptari. The PKC is not only characterized by the presence of protected areas such as PNP and KTWR but also consists of several community‐managed and unmanaged national forests. These forests play a vital role in conserving more than 50 mammalian species contributing to the region's rich biodiversity (Baral & Shah, 2008; Bhandari et al., 2015; DFRS, 2015; Jnawali et al., 2011; MoFE, 2021). In addition, PKC serves as an important corridor for Asian elephants (*Elephas maximus*), which migrate between Parsa and Koshi (Bhandari et al., 2022; Smith & Mishra, 1992).

The PKC is primarily covered by sub‐tropical forests, with sal (*Shorea robusta*) and mixed forests dominated by acacia (*Acacia catechu*) species (Chaudhary & Subedi, 2019; DFRS, 2015; Koirala et al., 2021; MoFE, 2021; Smith et al., 1998). These forests provide crucial habitat for various wildlife species, including sloth bears. They serve as a sanctuary for numerous plant and animal species, supporting the overall ecological balance of the region. The local communities residing in the PKC heavily rely on agriculture and livestock farming for their subsistence (DFRS, 2015; MoFE, 2021). Additionally, forest products such as firewood, leaves, and wood are harvested for various subsistence purposes (MoFE, 2021).

Despite its ecological significance, the PKC is facing multiple threats from human activities. One of the major challenges is deforestation in government‐managed forests (DFRS, 2015; Kafle et al., 2023; MoFE, 2021). Uncontrolled tree felling and illegal extraction of natural resources such as gravel, sand, and stones from the nearby hill regions are causing extensive damage to the landscape (Chaudhary & Subedi, 2019; DFRS, 2015). These activities not only disrupt the natural balance but also lead to adverse consequences such as floods, landslides, erosion, and the loss of wildlife species. Furthermore, ongoing human development projects pose significant threats to the PKC (Bhandari et al., 2015; DFRS, 2015; MoFE, 2021). The construction of highways, roads, railways, and buildings involves the destruction of natural forests, fragmenting habitats, and disrupting wildlife corridors (Bhandari et al., 2022; DFRS, 2015). These infrastructure projects, though aimed at promoting human development, can have adverse impacts on the local ecosystems and biodiversity.

Given the ecological importance of the PKC and the numerous threats it faces, comprehensive conservation strategies are essential to ensure its long‐term survival. It is crucial to establish a balance between human development and environmental protection, implementing sustainable practices that safeguard the landscape's rich biodiversity and the well‐being of local communities. Efforts should be made to promote responsible forest management, discourage illegal and unsustainable resource extraction, and create awareness about the value of conserving the PKC's natural resources. Collaboration between local communities, government authorities, and conservation organizations is vital to addressing the challenges and protecting the ecological integrity of the PKC.

### 2.2 Data collection

We collected the Sloth bear occupancy data between December 2022 and March 2023. A total of 152 camera traps (Stealth Cam STCG45NG) were stationed across the PKC for 3192 trap nights (152 sites × 21 days) by establishing 5 km × 5 km grids and deploying four cameras per grid. The interval between two camera traps was at least 1 km from each other. However, due to logistic constraints for regular monitoring, we did not deploy camera traps at the same time throughout the study area. The cameras were set to work for 24 h continuously during the survey across the forest habitats, and areas without forest were excluded from the study. Cameras were stationed at an average height of 40–60 cm above the ground focusing major tracks and trials used by wildlife, and each camera was set to capture three pictures with a burst delay of 30 s for second capture. Cameras were checked weekly and each week of the survey period was taken as a single sampling occasion across the study area, and only the data of mammal species detected were used for the study.

At each camera trapping station, we recorded habitat variables such as canopy cover, presence of large predators, number of humans detected, number of livestock detected, distance to a water body, distance to a settlement, and distance to major roads. A 10 m × 10 m plot was established at the camera trap station, keeping the camera as the center, and canopy cover was measured as an average of the four corners and the center. It was measured using the Gap Light Analysis Mobile Application (GLAMA; Tichý, 2014). The information on the presence of large predators as well as the number of humans and livestock detected was taken from the same camera trap. The distance to the nearest settlement, distance to the nearest water body, and distance to the nearest road were measured using measuring tape, but whenever the distance exceeded 200 m, it was measured using QGIS.

### 2.3 Data analysis

We used R program (R Core Team, 2023) to process and analyze the data implementing hierarchical occupancy modeling to identify correlates of sloth bear occupancy in PKC. The analysis was done following Royle and Dorazio (2008) creating an object data matrix for species detection at each site *i*, across each replicate survey. We used occupancy as an estimate of habitat selection for the analysis (Gould et al., 2019). First, the occupancy is given as,

$$z_{i}\sim \text{Bernoulli}\;\left(\psi \right)$$

where *z* is a latent variable that can be drawn from detection histories and *z*_*i*_ is drawn from a Bernoulli distribution with the parameter probability *ψ*. The detection probability however was modeled with the detection histories with a binomial distribution where, if *z*_*i*_−1, *p* is the probability of success, and if *z*_*i*_−1, the probability of success equals zero (*y*_*i*_ ~ Binomial (*n*_*i*_, *ps*_*i*_)). Where *i* is the number of sites and *n*_*i*_ is the number of replicates out of the total when the species is detected at each site *i*.

The variables taken for the study were tested to identify if there is any correlation among the predictors. The threshold for the correlation was set at |*r*| > .7. We observed that no predictors were highly correlated; therefore, we included all these predictors in the occupancy analysis. We used hierarchical occupancy modeling with logistic regression and logistic link function to gain insights on relationship between anthropogenic and ecological covariates and sloth bear occurrence. We used the detection probability of only two large carnivores; tiger and leopard collectively as the variable of large predators.

Since *ψ* is a probability of occupancy, the equation is given as,

$$\begin{array}{c} \text{logit}\;\left(\psi_{i}\right)=\beta_{0}+\beta_{\mathrm{cc}}{\mathrm{cc}}_{i}+\beta_{\text{livestock}}{\text{livestock}}_{i}+\beta_{\text{predators}}{\text{predators}}_{i}\\ \;+\beta_{\text{human}}{\text{human}}_{i}+\beta_{\text{settlement}}{\text{settlement}}_{i}+\beta_{\text{road}}{\text{road}}_{i}\\ \;+\beta_{\text{water}}{\text{water}}_{i} \end{array}$$

where *β*_0_ = logit (*ψ*_0_), an occupancy on logit scale and *β* varies for each species. We incorporated correlation between detection probability, occupancy, and intercept for the analysis (Devarajan et al., 2020).

We estimated model output implementing Markov Chain Monte Carlo (MCMC) simulation, and confirmed the convergence of model using Rhat value, with a threshold of 1.1. We used the adaptive MCMC using the jagsUI (Kellner et al., 2019), and coda (Plummer et al., 2006) packages in the R program and Just Another Gibbs Sampler (JAGS; Plummer, 2003) with three chains, 1000 adaptations, and 15,000 iterations. We derived a detection probability map (1 pixel = 1 km × 1 km) of sloth bear across PKC using inverse distance weighting (IDW) interpolation in QGIS 3.24.1 (Tisler) using the tool “IDW Interpolation.” The attribute for interpolation was the detection probability of the species across the 152 sites. The detection probabilities for each site were derived as a proportion of survey replicates in which the species is detected.

## 3 RESULTS

**FIGURE 2:**
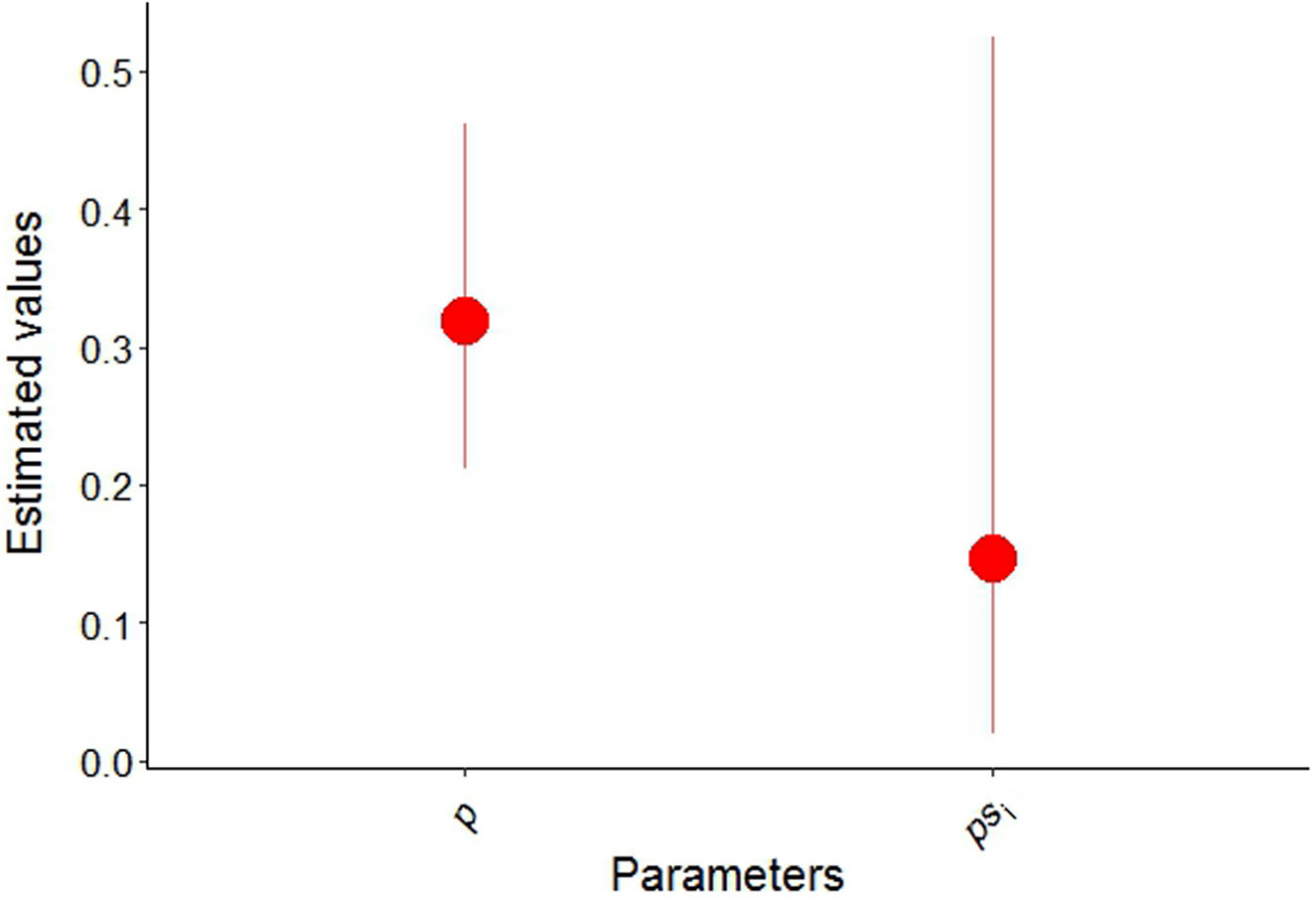
Detection probability (*p*) and occupancy (*ps*_*i*_) of sloth bear in Parsa–Koshi Complex in the lowland of Nepal.

We recorded a total of 46 detections of sloth bears across 30 of the total 152 sites. We measured a mean canopy cover 42.19 ± 21.36 (SD)% across our study sites. The mean numbers of human and livestock detections were 76.72 ± 244.55 and 36.74 ± 102.45, respectively. The mean distance to the nearest water body was 3354.83 ± 2213.63 (SD) m, distance to the nearest major road was 741.69 ± 1138.91 (SD) m, and the distance to the nearest settlement was 2182.80 ± 1691.33 (SD) m. We found the sloth bear has a modest detection probability (0.319 ± 0.065) with lower value of occupancy (0.15 ± 0.13) across PKC (Figure 2). However, the mean detection probability of other large carnivores (tiger and leopard) across the study sites was 0.37 ± 0.48.

**FIGURE 3:**
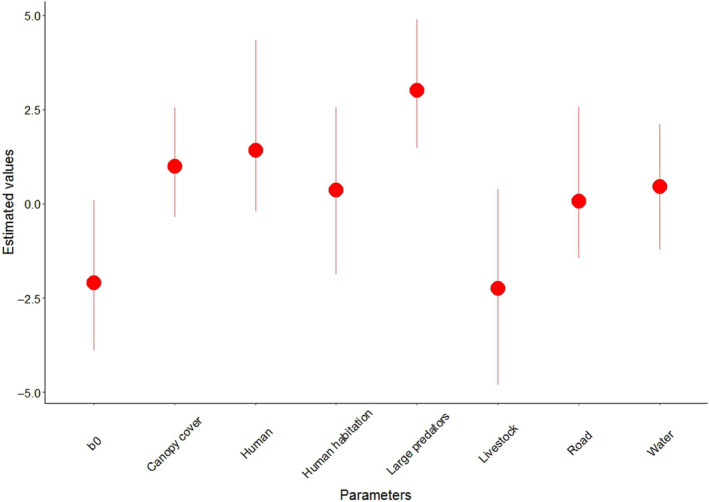
Estimated effect of covariates on sloth bear occurrence along with their upper and lower credible intervals in Parsa–Koshi Complex, in the lowland of Nepal.

We observed an increase in sloth bear occurrence with increase in all the studied variables except for the number of livestock detected (*βlivestock* = −2.24 ± 1.46) which interacted negatively (Table S1; Figure 3). The presence of large predators was observed to have positive influence on sloth bear occurrence (*βpredators* = 3.01 ± 0.96), followed by the number of humans detected (*βhuman* = 1.42 ± 1.21) and canopy cover percentage (*βcc* = 1.00 ± 0.73) (Table S1). On the contrary, the least positive interaction was observed for distance to the road (*βroad* = 0.06 ± 0.98), followed by distance to human settlements (*βsettlement* = 0.36 ± 1.07) and distance to water body (*βwater* = 0.46 ± 0.82).

**FIGURE 4:**
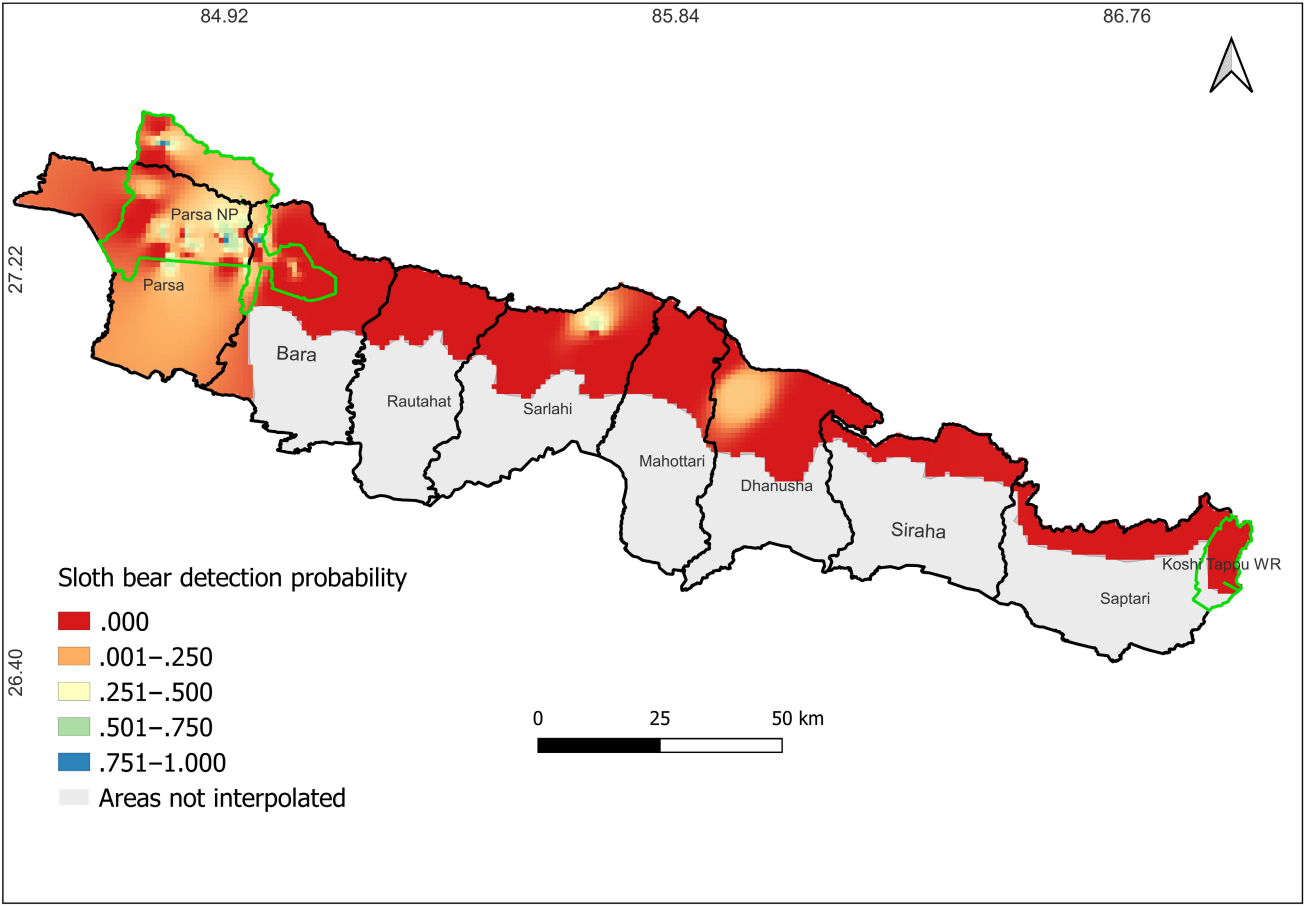
Inverse distance weighting (IDW) interpolation‐based detection probability map of sloth bear across Parsa–Koshi Complex in the lowland of Nepal.

We observed a higher detection probability of sloth bears in the western part of the PKC, mostly in the areas of Parsa and Bara districts (Figure 4). There are some places in the middle part of the PKC, mainly the Chure area of Sarlahi and Dhanusha districts, where there is a mild probability of the species detection. However, the eastern part of the study site close to KTWR shows zero detection probability for the species.

## 4 DISCUSSION

Our findings provide valuable insights into the ecological characteristics of the PKC and their implications for the detection probability and occupancy of sloth bears. We found the likelihood of detection of sloth bears was relatively moderate in PKC, which might be due to their secretive nature (Garshelis, Joshi, & Smith, 1999; Garshelis, Joshi, Smith, et al., 1999). In addition to our study, many other studies of the lowlands of Nepal also mentioned a modest detection probability of the sloth bear in the CNP and Chure region (Paudel et al., 2022; Pokharel et al., 2022), whereas there was a low detection probability in Mudumalai Tiger Reserve, Western Ghats, India (Ramesh et al., 2012), and Karnataka, India (Das et al., 2014). This is probably due to the rapidly declining global population density of sloth bears (Dharaiya et al., 2020). Similarly, the lower occupancy of the sloth bear in our study area might be due to lower species occurrences outside the protected areas, their low detection probability as well as low population densities (Garshelis, Joshi, & Smith, 1999; Garshelis, Joshi, Smith, et al., 1999). It was probably due to high disturbance, fragmentation, and rapid exploitation of forest resources, which accelerates degradation of the suitable habitats of sloth bears. These degraded habitats outside the protected areas possess the low termite build density (H. P. Sharma, personal observation), as termite is a major prey species for sloth bears. The majority of the studies inside the protected areas show higher occupancy (Puri et al., 2023; Ramesh et al., 2012).

In the western part of the PKC, we observed a higher detection probability of sloth bears. However, in the central and eastern parts of this complex, there was low to zero detection probability for the species. This discrepancy could be attributed to the presence of PNP, a protected area where sloth bear distribution is likely to be higher. Our results also indicated a higher detection probability of sloth bears in the buffer zone community forests and some other community forests near PNP. These protected areas ensure better conservation and management of wildlife compared with non‐protected areas. It is surprising that there is almost no detection of sloth bears in most parts of the central and eastern regions, despite the potential suitability of those habitats for the species. This raises questions about the presence of other large predators in those areas. However, previous study conducted by Bhandari et al. (2015) have reported the presence of large predators, such as leopards and hyena (*Hyaena hyaena*) in this landscape. Nonetheless, anthropogenic pressure and negative attitudes toward wildlife conservation by local communities can contribute to the decline of carnivore populations (Baral et al., 2022). The eastern region is subject to high human disturbance with extremely degraded and fragmented Churia forests, a prime habitat of the species in Nepal (Hari Prasad Sharma). This almost zero or low detection probability of sloth bears in the eastern and central regions, which are located outside protected areas, suggests potential threats to the species in the future as well (Baral & Shah, 2008; Jnawali et al., 2011).

We found positive relationship between sloth bear occurrence with all the variables, such as the presence of large predators, forest canopy cover, and the nearest distance to water sources. Humans, distance to settlements, and distance to roads also exerted positive impact on sloth bear occupancy, except for the number of livestock detected, which negatively interacted with sloth bears. The presence of sloth bears is influenced by the presence of large predators, such as tigers, leopards, which might reduce resource competition among the herbivores, and providing protection from other potential threats (Carter & Linnell, 2016; Gulati et al., 2021; Puri et al., 2020; Ripple et al., 2014; Wolf & Ripple, 2018). The positive interaction between these carnivores and sloth bear occurrence indicates the existence of a healthy prey base for tigers and leopards as well as termites for sloth bear in the area, which is essential for their survival (Carter & Linnell, 2016; Ford & Goheen, 2015; Ripple et al., 2014; Sillero‐Zubiri & Laurenson, 2001; Widodo et al., 2022). For example, tigers and leopards primarily prey on ungulates (Wegge et al., 2009), and their presence suggests the availability of suitable habitat such as open habitat, grassland, and forest (Ofstad et al., 2016), and these suitable and pristine habitat is also home to the large number of termite build, fruits, and prey resources (Benzie, 1986; Joshi et al., 1997) that can sustain sloth bears as well.

The canopy cover across our study sites indicates moderate vegetation density and its variation within the landscape. The occupancy of sloth bear is supported by the increased canopy cover percentage, that might be due to the bear's preference for forested habitats. However, the lower occupancy is probably due to moderate vegetation density and variation within the landscape. Generally, they prefer higher forest cover and topographic heterogeneity (Chaudhuri et al., 2022; Puri et al., 2015; Srivathsa et al., 2018). The sloth bears are adapted to forest environments, relying on trees for shelter, nesting, and foraging (Chaudhuri et al., 2022; Srivathsa et al., 2018). Higher canopy cover can provide suitable habitat conditions and protection for sloth bears, contributing to their increased occupancy in areas with dense tree cover.

Furthermore, we assessed the positive association of sloth bear occupancy with water body, which indicates the availability of water sources within the landscape, which can be a crucial factor for the survival and distribution of sloth bear. In addition, the moist soil conditions near water bodies support a high abundance of termites, the key food source for sloth bears (Ratnayeke et al., 2007). The well‐drained soft soils surrounding water bodies also facilitate easier foraging for termites (Akhtar et al., 2004). However, the occupancy probability of sloth bear in this study increased with increasing the water source distance, these areas might have abundant suitable foraging and denning habitat that makes these sites attractive to bears (Akhtar et al., 2004; Bashir et al., 2018; Benson & Chamberlain, 2007; Jain et al., 2021). Therefore, sloth bears might exhibit a preference for sites to perennial water sources (Pokharel et al., 2022).

The positive association between sloth bear occurrence and the number of humans detected and the nearest distance to settlements might be due to the sloth bear's opportunistic feeding behavior (Akhtar et al., 2004). They scavenge the human waste and raid crops, which are readily available in human‐dominated areas (Seidensticker et al., 2011). Therefore, higher human densities may provide additional food sources for sloth bears, and increases their occurrence in these areas. The closeness of sloth bear with human sometimes increases their attack on human (Singh et al., 2018) as they are found in both protected and non‐protected forests, including multi‐use and reserve forests (Baral & Shah, 2008; Pokhrel et al., 2022). Forests with human influence can serve as corridors for movement between protected areas, benefiting large mammal species (Athreya et al., 2013; Puri et al., 2015). Sloth bears often overlap with areas of high human populations (Akhtar et al., 2004). However, those scenarios can contribute to increasing the human–sloth bear interface and cause conflicts.

Our study suggests sloth bear avoided the areas with high number of livestock detection probably due to competition with livestock (Akhtar et al., 2004; Puri et al., 2015). Sloth bears are omnivorous, and their diet consists of a variety of food sources, including fruits, insects, and small mammals (Joshi et al., 1997; Seidensticker et al., 2011). As the number of livestock increases in an area, it may lead to increased competition for resources, including food, or conflicts with livestock owners (Silwal et al., 2017), resulting in a negative impact on sloth bear occurrence (Rajpurohit & Krausman, 2000). Additionally, instances of retaliatory killings by livestock owners in response to sloth bear attacks on their livestock can further contribute to the negative interaction.

## 5 CONCLUSIONS

Our study specifically focused on the detection probability and occupancy of the sloth bear in the PKC. We found that the sloth bear exhibited a modest occupancy, suggesting that detecting this species in this area can be challenging. Additionally, we estimated a lower occupancy for the sloth bear, indicating that the presence and distribution of this species within the study area were relatively limited. This study also highlights the intricate relationships between sloth bears, humans, large predators, and livestock, underscoring the importance of comprehensive conservation strategies that consider ecological and socio‐economic factors. This approach is essential to ensure the long‐term survival of sloth bear populations in the lowland Nepal. Our study also assumes that the lack of conservation approaches in the eastern and central parts of the study area may be responsible for the almost nonexistent or low detection of sloth bears in those regions. This is primarily due to the gaps of protected areas between the PKC in the lowland. Consequently, we recommend the implementation of conservation measures in the areas between Parsa and Koshi, as this would benefit not only sloth bears but also many threatened species inhabiting the lowland of Nepal.

## AUTHOR CONTRIBUTIONS

**Hari Prasad Sharma:** Conceptualization (equal); data curation (equal); formal analysis (equal); funding acquisition (equal); investigation (equal); methodology (equal); project administration (lead); supervision (lead); writing – original draft (equal); writing – review and editing (equal). **Hem Bahadur Katuwal:** Conceptualization (equal); data curation (equal); funding acquisition (equal); investigation (equal); methodology (equal); writing – review and editing (equal). **Bishnu Prasad Bhattarai:** Conceptualization (equal); data curation (equal); funding acquisition (equal); investigation (equal); methodology (equal); writing – review and editing (equal). **Shivish Bhandari:** Investigation (equal); methodology (equal); writing – original draft (equal); writing – review and editing (equal). **Dipendra Adhikari:** Investigation (equal); writing – review and editing (equal). **Bishnu Aryal:** Investigation (equal); writing – review and editing (equal). **Krishna Tamang:** Investigation (equal); writing – review and editing (equal). **Amrit Nepali:** Investigation (equal); writing – review and editing (equal). **Sabin KC:** Investigation (equal); writing – review and editing (equal). **Bashu Dev Baral:** Investigation (equal); writing – review and editing (equal). **Surya Devkota:** Investigation (equal); writing – review and editing (equal). **Sabina Koirala:** Funding acquisition (equal); writing – review and editing (equal). **Dev Narayan Mandal:** Data curation (equal); writing – review and editing (equal). **Sandeep Regmi:** Conceptualization (equal); data curation (equal); formal analysis (equal); investigation (equal); writing – original draft (equal); writing – review and editing (equal).

## CONFLICT OF INTEREST STATEMENT

The authors declare no conflicts of interest.

## Supporting information


Table S1

## Data Availability

The data are available on dryad DOI: 10.5061/dryad.2ngf1vhv9.
