# Supplementary material for: Factors affecting the occupancy of sloth bear and its detection probability in Parsa–Koshi Complex, Nepal
Source: Ecol Evol. 2023 Oct 3;13(10):e10587. doi: 10.1002/ece3.10587 (PMC10547580; doi:10.1002/ece3.10587)
Supplement: Supplementary file 1 — Table S1 [file ECE3-13-e10587-s001.docx]

Supplementary Table 1: Detection probability (p), occupancy (psi) and relationship of different anthropogenic and ecological factors on sloth bear occupancy. SD: standadrd deviation, LCI: lower credible interval, UCI: upper credible interval, Rhat, n.eff: effective sample size, overlap0: Bayesian credible overlap, f: proportion similar to prior.

| **Parameters** | **Mean** | **SD** | **LCI** | **Md** | **UCI** | **Rhat** | **n.eff** | **overlap0** | **F** |
| --- | --- | --- | --- | --- | --- | --- | --- | --- | --- |
| p | 0.319 | 0.065 | 0.212 | 0.311 | 0.462 | 1.004 | 540 | 0 | 1 |
| psi | 0.147 | 0.132 | 0.020 | 0.100 | 0.524 | 1.020 | 200 | 0 | 1 |
| b0 | -2.096 | 1.016 | -3.887 | -2.198 | 0.096 | 1.010 | 254 | 1 | 0.969 |
| Canopy Cover (%) | 1.002 | 0.737 | -0.358 | 0.971 | 2.550 | 1.001 | 2260 | 1 | 0.922 |
| Livestock (n) | -2.240 | 1.467 | -4.807 | -2.218 | 0.387 | 1.001 | 2866 | 1 | 0.939 |
| Large predators (presence) | 3.014 | 0.968 | 1.484 | 2.854 | 4.886 | 1.007 | 326 | 0 | 1 |
| Distance to water (m) | 0.463 | 0.825 | -1.229 | 0.469 | 2.103 | 1.002 | 880 | 1 | 0.737 |
| Human (n) | 1.428 | 1.216 | -0.211 | 1.160 | 4.348 | 1.001 | 6347 | 1 | 0.933 |
| Distance to road (m) | 0.066 | 0.981 | -1.441 | -0.114 | 2.579 | 1.006 | 684 | 1 | 0.438 |
| Distance to human habitation (m) | 0.362 | 1.074 | -1.862 | 0.371 | 2.545 | 1.005 | 444 | 1 | 0.660 |
